# 82 Comparison of Parent vs. Burn-Injured Youth Scores on the Strengths and Difficulties Questionnaire

**DOI:** 10.1093/jbcr/irad045.056

**Published:** 2023-05-15

**Authors:** Ruth Rimmer, Curt Bay, Emile Kalil, Daniel Chacon, Erika Mendoza, Kevin Foster

**Affiliations:** Arizona Burn Center Valleywise Health, Phoenix, Arizona; AT Still University, Phoenix, Arizona; Mid-Atlantic Burn Camp Fund, Inc., Highland, Maryland; Alisa Ann Ruch Burn Foundation, Pasadena, California; Arizona State University, Phoenix, Arizona; AZ Burn Center at Valleywise Health, Cave Creek, Arizona; Arizona Burn Center Valleywise Health, Phoenix, Arizona; AT Still University, Phoenix, Arizona; Mid-Atlantic Burn Camp Fund, Inc., Highland, Maryland; Alisa Ann Ruch Burn Foundation, Pasadena, California; Arizona State University, Phoenix, Arizona; AZ Burn Center at Valleywise Health, Cave Creek, Arizona; Arizona Burn Center Valleywise Health, Phoenix, Arizona; AT Still University, Phoenix, Arizona; Mid-Atlantic Burn Camp Fund, Inc., Highland, Maryland; Alisa Ann Ruch Burn Foundation, Pasadena, California; Arizona State University, Phoenix, Arizona; AZ Burn Center at Valleywise Health, Cave Creek, Arizona; Arizona Burn Center Valleywise Health, Phoenix, Arizona; AT Still University, Phoenix, Arizona; Mid-Atlantic Burn Camp Fund, Inc., Highland, Maryland; Alisa Ann Ruch Burn Foundation, Pasadena, California; Arizona State University, Phoenix, Arizona; AZ Burn Center at Valleywise Health, Cave Creek, Arizona; Arizona Burn Center Valleywise Health, Phoenix, Arizona; AT Still University, Phoenix, Arizona; Mid-Atlantic Burn Camp Fund, Inc., Highland, Maryland; Alisa Ann Ruch Burn Foundation, Pasadena, California; Arizona State University, Phoenix, Arizona; AZ Burn Center at Valleywise Health, Cave Creek, Arizona; Arizona Burn Center Valleywise Health, Phoenix, Arizona; AT Still University, Phoenix, Arizona; Mid-Atlantic Burn Camp Fund, Inc., Highland, Maryland; Alisa Ann Ruch Burn Foundation, Pasadena, California; Arizona State University, Phoenix, Arizona; AZ Burn Center at Valleywise Health, Cave Creek, Arizona

## Abstract

**Introduction:**

Research regarding the prevalence of psychopathology issues in burn-injured youth is limited. The Strength and Difficulties Questionnaire (SDQ) is valid for both self- and parent-report, and is widely used. This study sought to evaluate the level of agreement between the youth self-report and parental response.

**Methods:**

Respondents included 36 adolescent/parent dyads. The youth included 11-18 years old, 14% African American, 19% Hispanic and 44% White, 19 females, 17 males with 58% reporting both visible & hidden scars. The SDQ is a widely used behavioral screening tool assessing children’s positive & negative attributes on 5 scales: 1) Emotional Symptoms, 2) Conduct Problems, 3) Hyperactivity/Inattention, 4) Peer Problems, 5) Prosocial Behavior as well as a Total Difficulties Score.

**Results:**

Youth reported significantly higher scores than parents on Total Difficulty (*P* < 0.001). They had significantly higher scores on all scale scores – Emotional Problems (*P* = 0.01), Hyperactivity (*P* = 0.001), Conduct Problems (*P* = 0.041), Peer Problems (*P=0.041*), Internalizing (*P* = 0.005), and Externalizing Behaviors ((*P* < 0.001), but not the Pro-Social Scale (P=0.303). Correlations between self- and parent-report were significant only for the Hyperactivity scale, r=.40, p=0.017.

**Conclusions:**

The large difference in youth vs. parent responses is of concern because study parents appear to be unaware of psychosocial issues challenging their children. This study mirrors other research which has documented a lack of awareness of parents regarding children’s reported anxiety disorder symptomology.

**Applicability of Research to Practice:**

Healthcare providers & parents should discuss burn-injured youths’ feelings regarding their progress. Providers should not rely solely on parents when determining the well-being of pediatric survivors. This survey can be completed easily by youth and parents in the aftercare setting and used as a tool for recommending intervention when difficulties are present.

